# Music therapy as social skill intervention for children with comorbid ASD and ID: study protocol for a randomized controlled trial

**DOI:** 10.1186/s12887-020-02454-6

**Published:** 2020-12-05

**Authors:** Yen Na Yum, Way Kwok-Wai Lau, Kean Poon, Fuk Chuen Ho

**Affiliations:** Department of Special Education and Counselling, The Education University of Hong Kong, 10 Lo Ping Road, Tai Po, New Territories, Hong Kong SAR, China

## Abstract

**Background:**

Autism spectrum disorder (ASD) is a developmental impairment characterized by persistent deficits in social communication and interactions, and over half of children with ASD possess below average intellectual ability (IQ < 85). The social development and response to social skill interventions among children with ASD and comorbid intellectual disability (ID) is not well understood. Music therapy is a systematic process of intervention, wherein a therapist may help clients promote their social skills by using musical experience. The proposed study will address limited research evidence on music therapy as an intervention for social functioning in children with ASD with mild to borderline ID.

**Method:**

A randomized controlled trial (RCT) with two parallel groups of 40 children each (1:1 allocation ratio) is planned. Participants will receive 45 min of music therapy or non-musical intervention targeting social skills once a week for 12 weeks. Primary outcome measures will be independent ratings on the Childhood Autism Rating Scale and parent ratings on the Social Responsiveness Scale-2. Linear mixed-effects models for these two outcome measures will be created for data collected at 2-week pre-intervention, 2-week post-intervention, and 4-month post-intervention sessions. In-session behaviors at the first and last intervention will be videotaped and coded offline and compared. Pretreatment neural response of quantitative electroencephalograms (qEEG) to social scenes will be used to predict the outcomes of musical and non-musical social skill interventions, whereas qEEG responses to music will be used to predict the effectiveness of musical social skill intervention.

**Discussion:**

If neural markers of social skill development are found, then the long-term goal is to develop individualized intervention based on pre-treatment markers to maximize treatment efficacy. The proposed study’s results may also suggest directions to development and provision of music therapy services in Hong Kong.

**Trial registration:**

ClinicalTrials.gov (NCT04557488). Registered September 21, 2020.

## Background

Autism spectrum disorder (ASD) is a developmental impairment characterized by persistent deficits in social communication and interactions coupled with restricted and repetitive patterns of behaviors. The symptoms of ASD appear in early childhood. The prevalence of childhood ASD in the Greater China Region is 26.6 per 10,000 children [1], but the figures have increased rapidly in recent years due to the expansion of diagnostic criteria and increased public awareness. Given that no known cure or effective pharmacological intervention for ASD is available, behavioral interventions that target autism symptoms is the main strategy to support individuals with ASD to be integrated into school and the society. Intervention studies typically target the core social deficits in ASD, including low inclination for human interactions, lack of social skills, and impairments in interpersonal communication. Social skill training may cover a myriad of social behavior, including initiating interaction, responding, maintaining eye contact, using appropriate co-speech gestures, and conversational turn-taking. Training for high-level social cognition may involve understanding non-literal language, perspective taking, and recognizing emotions [2].

Although high-functioning ASD individuals do not tend to naturally comprehend social conventions, they can follow established rules and imitate modeled social behavior. However, the effectiveness of explicit social skill training may not generalize to low-functioning school-aged children with ASD. Explicit training is likely to be less effective for children with severe autistic symptoms who actively avoid social interactions or who may demonstrate limited intellectual ability. The clinical presentations of ASD vary to a large extent, and approximately 56% of children with ASD possess below average intellectual ability (IQ < 85) [3]. The intellectual, verbal, and social ability in this population may greatly influence intervention outcomes. The social development of children with ASD and comorbid intellectual disability (ID) is not well understood, and systematic investigations on how children with ASD and ID respond to social skill interventions remains to be done.

### Use of music in social skill intervention

The choice to examine music in social skill intervention is motivated by accruing evidence of its potential for therapeutic use for various clinical populations [4]. The use of music in ASD intervention is quite common. Background music may be used to increase familiarity and relaxation, whereas active music activities can provide a point of contact between instructors and children with ASD, thereby helping to build rapport and promote learning through non-verbal interactions [4, 5]. However, music therapy as social skill intervention for low-functioning ASD individuals have not been comprehensively examined. For ASD intervention, music has been explored as a medium to increase social skills in the field of music therapy for some years [5]. Music therapy is defined as a systematic process of intervention, wherein a therapist helps clients promote their health by using musical experience and relationships that develop through them [6]. Children with ASD and ID are often described as preoccupied in their own world and may be highly passive or unresponsive in social situations. The low verbal ability of these children is one limitation to their participation given that social interactions often involve verbal exchanges. Music may become a conduit for children whose natural affinity to social interactions is impaired because group music making and improvisations encourage initiation and turn-taking behavior in a non-verbal situation. In group music therapy, children with ASD can learn to tolerate the presence of and physical contact with other people, distinguish between oneself and others, and practice social behavior [5, 6].

A Cochrane review in 2014 analyzed behavioral data from 10 RCT studies comparing music therapy with non-musical placebo therapy or standard care [7]. The results demonstrated moderate-quality evidence supporting the effectiveness of music therapy in improving social interactions and initiating behavior, social adaptation, and parent–child relationship. No adverse effects have been reported. Although flaws in study designs and low numbers of participants in some studies have precluded strong conclusions, available evidence indicates that music therapy improves social–emotional reciprocity and verbal and nonverbal communicative skills outside the context of therapy. By contrast, a recent large-scale multicenter RCT study on music therapy for 364 children with ASD aged 4–7 years has found no significant differences in social skills compared with standard care as measured by clinical observations or parental ratings [8]. These mixed findings may be due to the heterogeneity of the studies, which involved children belonging to different age groups, abilities, and living in different countries. Long-term outcomes are also unclear in these studies. Since effectiveness of clinical interventions is influenced by social and cultural contexts [6], previous studies must be replicated and extended to obtain locally relevant data. The proposed study will contribute to a clear evidence base that serve to inform and develop music therapy in social skill interventions for children with ASD and ID in Hong Kong.

### Prediction of treatment success

One hypothesis that has not been tested in previous studies is that success in social skill interventions may depend on individual differences. Autistic symptoms and abilities vary widely among children with ASD, arising both from different developmental speeds and specific interests. Some individuals may possess deficits in the areas of restricted interests or repetitive behavior, which results in poor social skills; however, they are accepting of social interactions. The willingness to seek out social experiences is the foundation to social skill development because without initial interests in social behavior, the motivation to attend to the explicit training and modeling is low. In such cases of social avoidance, cultivation of social interests through other strategies, such as music making, may be more effective. Narrowing down the characteristics of children who respond well to a particular type of intervention can facilitate timely and efficient treatment provision. To build the foundation for an evidence-based strategy for individualizing treatment plans, research should explore markers that indicate responsiveness to interventions. In this study, we propose to test quantitative electroencephalogram (qEEG) signals in response to music and social scenes as predictive measures of treatment outcomes.

Spontaneous electrophysiological neural activities at rest or when engaged in task can be measured by qEEG. Spectral analysis is used to decompose qEEG signals across several minutes into different frequency bands, which are associated with specific functions. The most consistent pattern of findings in the literature is that individuals with ASD show a U-shaped pattern of spectral power relative to controls without ASD [9]; that is, excessive power is observed at low-frequency (delta, 1-3 Hz and theta, 4–7 Hz) and high-frequency (beta, 13-35 Hz and gamma, > 35 Hz) bands, but reduced power is detected in middle-range frequency band (alpha, 8-12 Hz). The pattern was found with wide topographic distribution, which suggested abnormalities across multiple brain regions. This finding was reported for children in different age groups and for individuals with or without comorbid ID. High-frequency bands have been associated with emotional responses and emotion recognition and may be linked to such deficits in individuals with ASD. Neural patterns that deviate from age-matched controls to a greater extent may suggest more severe behavioral symptoms.

Frontal alpha asymmetry (FAA) is typically investigated in relation to emotional response and motivations, both in clinical and normal populations [10]. Left-lateralized or left-dominant brain activity has been linked to an *approach* system where an individual experiences positive emotions and motivations. In contrast, right-lateralized frontal activity may reflect negative emotions and intention to withdraw. Reduced alpha power in the left frontal area of the scalp in individuals with ASD has also been reported [9]. The modulation of FAA has been demonstrated in neurofeedback training in individuals with ASD to activate their imitation behaviors [11, 12], so this measure is hypothesized to predict and be responsive to social skill interventions. High-functioning individuals with ASD show intact emotional processing when listening to music, although they demonstrate distinct neural patterns relative to neurotypical adults, which is interpreted as increased cognitive load and physiological arousal [13]. Individual preference for music varies within the target group with ASD and ID and may predict how they respond to social skill intervention using music therapy. Although self-report rating scales exist for reporting music experience and preference, qEEG measures may be superior because they index automatic responses to musical stimuli that the target population of children with ASD and ID may find difficult to understand or express.

The proposed study will address the research gap on music therapy as an intervention for social functioning in children with ASD with mild to borderline ID. The research design is to examine whether using music therapy as social skill intervention provides additional benefits relative to non-musical active control behavioral intervention in a 12-week RCT. We will also evaluate whether pre-treatment neural response to music is correlated with the effectiveness of musical social skill intervention. If correlation is found, then the long-term goal is to develop individualized intervention based on pre-treatment markers to maximize treatment efficacy.

### Objectives

Aims and hypotheses to be tested:

1. On the group level, is social skill intervention using music therapy more effective in enhancing social interaction than non-musical social skill training for children with ASD and co-occurring mild/borderline ID?
2. On the individual level, are participants with enhanced left-lateralized FAA to social scenes relative to baseline more responsive to both types of social skill interventions?
3. For participants in the music therapy intervention, does enhanced left-lateralized FAA to preferred music relative to baseline predict better outcomes in social skill development?

## Methods/design

### Study design

**Fig. 1:**
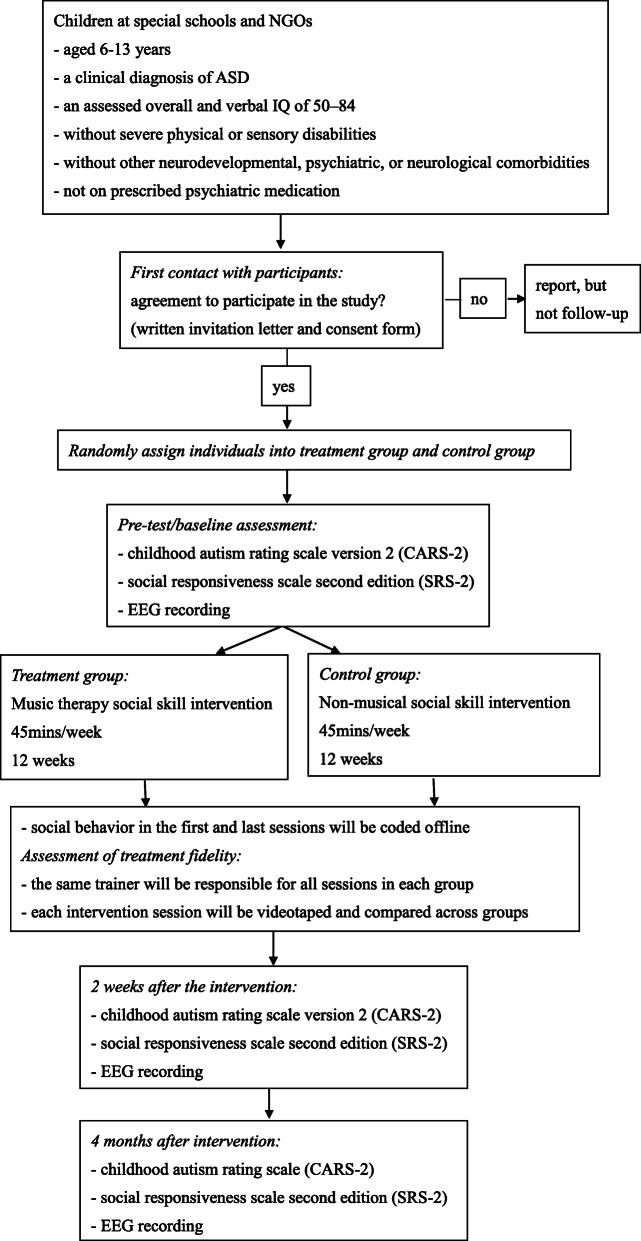
Study design and participant enrolment

The proposed study will be a randomized controlled trial with two parallel groups. Recruited children will first be screened for eligibility and then matched into pairs by age and gender by a research assistant. Another research assistant will assign individuals in each pair to the treatment group (social skill intervention with music) or control group (non-musical social skill intervention) using random number sequences. Participants in the same condition will then be separated into groups of eight by drawing lots. The research assistants performing participant matching and randomization will not be involved in data collection or outcome assessments. The pre-tests and post-tests will include (i) childhood autism rating scale-second edition (CARS-2) rated by an independent rater, (ii) social responsiveness scale-second edition (SRS-2) rated by parents, and (iii) EEG recording. Pre-tests will be administered 2 weeks before intervention, while post-tests will be administered 2 weeks and 4 months after intervention. In each intervention week for 12 weeks, the participants will undergo one 45 min session of social skill training. Within each condition, the same trainer will be responsible for all sessions to minimize differences in intervention delivery across groups. The intervention sessions will be videotaped, and social behavior in the first and last sessions will be coded offline. Figure 1 shows an overview of the study design. Should protocol modifications become necessary, the amendments will be communicated to relevant parties, including research personnel, intervention trainers, participants and their parents, the trial registry, the journal that published the study protocol, and the funding body.

### Participants

A total of 80 primary school aged children (aged 6–13 years) with ASD and mild/borderline ID will be recruited from various sources, including local special schools, music therapy centers, parent groups, and non-governmental organizations (NGOs) in Hong Kong. A research assistant will obtain written informed consent and oral assent from parents and participants, respectively, before participants enroll into the interventions. Participants and their families’ personal information will be kept strictly confidential and stored securely. Participants and families will receive interventions free of charge and stipends for transportation to the intervention and assessment sites. Participants will also be given souvenirs after pre- and post-intervention assessments as incentives.

### Inclusion and exclusion criteria

The inclusion criteria include a formal clinical diagnosis of ASD and an assessed overall and verbal IQ of 50–84 by a certified clinician. Children who report hypersensitivity to sounds will be included with consent but will be withdrawn immediately if adverse reactions that cannot be controlled (e.g., screaming, attempts at self-harm) are observed. Intervention trainers, assistants, or parents of participants will be encouraged to report any adverse events to facilitate timely response.

Children who exhibit severe physical or sensory disabilities (e.g., deafness) that may limit their participation in either intervention will be excluded. Children with other neurodevelopmental, psychiatric, or neurological comorbidities or are on prescribed psychiatric medication will also be excluded from the study. Children who already engage in music therapy or other social skill interventions (excluding standard care) will be ineligible to enroll in the study.

### Sample size calculation

The sample size was calculated based on the predicted effect size of 0.71 in generalized social interaction outside intervention context from a meta-analysis of social skill intervention studies using music therapy for children with ASD (aged 2–12) relative to non-music intervention or standard care controls [7]. For a two-group *t* statistics with α = 0.05, power = 0.8, and attrition rate = 25%, a minimum number of 33 participants in each group will likely yield statistically meaningful differences. Given that neural measures can detect autonomic responses and behavioral intentions in addition to explicit actions, they can be considered more change sensitive than behavioral observations. Hence, we expect that recruiting 40 participants in each group will allow us to detect presence of effects in qEEG measures.

### Interventions

#### Treatment group intervention

##### Trainers and participants

The treatment group will receive social skill intervention using music therapy in groups of eight. A certified music therapist with prior experience with children with ASD and ID will be the trainer for the treatment group. Parents or primary caregivers will be invited to attend the intervention sessions and to observe the training. An assistant trainer will also be present in all sessions to facilitate the group activities, manage unexpected situations, and ensure the safety of the participants.

##### Schedule and setting

The intervention will be delivered one session per week over a 12-week period. The sessions are 45 min in duration and are designed to be short because children with ASD and ID tend to exhibit a short attention span. The location will either be at a special schools or NGO in Hong Kong.

##### Session structure

Each session will follow a similar structure with a hello song, musical activities, and a goodbye song. The musical activities will vary in each session and will be mixed in later sessions to revisit and practice social skills [6].

##### Intervention contents and target skills

- Session 1–2: Familiarize the participants with the surroundings and other participants and the music therapy session structure using visual display; introduce the hello and goodbye songs
- Sessions 3–4: Increase vocalization and verbal use by song singing of familiar songs and social songs
- Sessions 5–6: Encourage communication of choice, sharing, and social interaction by selecting, using, and exchanging musical instruments
- Sessions 7–8: Emphasize group participation and sending and receiving social cues by group drumming and music making, and other coordinated movements during songs
- Sessions 9–10: Increase initiation, response, and turn-taking by free or structured improvisations of vocal and instrumental music
- Session 11–12: Review and reinforce past musical activities, appreciate and reward improvements, forewarn the end of intervention sessions

##### Emphasis of approach

The trainer will typically focus on building a relationship with the participants, understanding their current levels of functioning, and choosing musical activities that may scaffold their social development.

#### Control group intervention

##### Trainers and participants

The control group will receive behavioral-based social skill training in groups of eight. The trainer will be a registered social worker with experience in providing social skill training for children with ASD and ID. Parents or the primary caregivers will be invited to attend the intervention sessions and to observe the training. An assistant trainer will also be present in all sessions to provide support.

##### Schedule and setting

The intervention schedule will closely follow the treatment group’s schedule, with one 45-min session per week for 12 weeks. The location will either be at a special school or NGO in Hong Kong, using the same venue if feasible.

##### Session structure

Each intervention session will follow a standard structure of opening greetings, social activities according to the theme of the session, and a closing activity. The activities and games will vary in each session and will be mixed in later session to revisit and practice social skills [14].

##### Intervention contents and target skills

- Session 1–2: Familiarize the participants with the surroundings and other participants and the intervention session structure using visual display; introduce greetings and gestures when meeting new people and making friends via visual and video modeling
- Sessions 3–4: Increase vocalization and verbal use by playing pair and group games and small craft projects that require collaboration and communication
- Sessions 5–6: Encourage communication of choice, sharing, and social interaction by reading social stories and playing cooperative games
- Sessions 7–8: Emphasize group participation and sending and receiving social cues by role-play; exercises in recognizing emotions and display of appropriate emotions and behaviors in different daily settings
- Sessions 9–10: Increase initiation, response, eye contact, and turn-taking in conversation by explicit instructions or demonstrations, small group practice with feedback
- Session 11–12: Review and reinforce past activities and taught social skills, appreciate and reward improvements, forewarn the end of intervention sessions.

##### Emphasis of approach

Focus is typically on explicit and concrete instructions, reinforcing good social habits, and fostering positive associations for group interactions. Musical activities will be excluded in this control condition to contrast the specific effects of music therapy in intervention. This type of group training is typically offered by schools or NGOs in Hong Kong for children with ASD with or without ID, and may be considered as a standard practice control condition relative to music therapy targeting social skills.

### Assessment of treatment fidelity

Before intervention commences, the intervention manual will be shared with the trainers to facilitate their preparation and delivery of intervention. All materials and procedures that have been developed will be clearly described and explained in pre-intervention meetings with trainers. Adherence to the protocol and trainer competence will be directly assessed by the recorded video data and by independent review of the intervention session notes. The inter-trainer reliability and evaluation of treatment integrity will be reported in the outcomes. Participants will be asked for reasons for discontinuation or deviation from intervention protocols.

### Outcome measures

#### Primary outcomes

The primary outcome measure is autistic severity as assessed using a standardized rating scale by an independent rater who will be blinded to the study group allocation. This outcome was selected because observing behavioral changes that generalize to other social contexts outside of the intervention is the ultimate goal of social skill intervention. Parental rating by using a standardized rating scale is included as another outcome to support the clinical assessment. Parents are good informants because they spend time with the participants outside of intervention contexts, but they are not blinded to the group assignment and may exhibit expectation biases. Another outcome is the behavioral changes at the last intervention sessions relative to the first session. Trained research assistants who are blinded to the session number and study hypotheses will code for target social behaviors by using video data. This measurement will provide an objective measure of the social skills exhibited by participants within the intervention setting.

##### CARS-2

The general autistic symptoms of the participants will be assessed using CARS-2 [15], by a qualified and experienced assessor. This scale consists of 15 questions rating the autistic symptoms and general impression of deviance based on behavioral observations. Each question is rated from 1 to 4, and high scores are associated with a high level of impairment. Scores below 30 indicate that an individual does not have ASD, scores between 30 and 36.5 imply mild to moderate autism, and scores from 37 to 60 correspond to severe autism. The psychometrics of CARS-2 is well documented in Western and Chinese contexts [15, 16].

##### SRS-2

Parental ratings of generalized social interaction outcomes will be collected using SRS-2. A parent of each participant will be asked to complete the SRS-2 based on their observations of their child over the previous 3 months. The scale is a 65-item questionnaire that measures the severity of social impairments associated with ASD [17]. The five subscales include social awareness, social cognition, social motivation, social communication, and autistic mannerisms. Each item is rated on a scale from “0” (never true) to “3” (almost always true), and high scores indicate severe social impairments. SRS-2 exhibits good psychometric properties in Western and Chinese contexts and is sensitive to changes in social functioning among children with ASD [17, 18].

##### In-session social behavior

The intervention sessions in the treatment and control groups will be videotaped using a high-resolution video camera, and data from the first and last sessions will be coded for target social behavior. The three main types of behavior, namely, initiation, turn taking, and eye contact, will be examined using a microanalysis of video recordings in one-second intervals [19]. Target social skills were selected based on the expected types of behavior that may occur during the sessions and that are essential for effective social interactions. The frequency and duration of the social behavior will be recorded with the following coding scheme. Initiation includes touching a person, making a verbal request, giving an object to a person, and other actions to get another’s attention. Turn taking includes responding within an appropriate time either verbally or non-verbally and not interrupting out of turn. Eye contact includes directing the gaze toward the person speaking or acting. The behavior will be further categorized as instructor-to-participant social interactions or participant-to-participant social interactions. Inter-rater reliability will be determined in 20% of coding. These behavioral outcomes are important indices of how much and how appropriately children engage in social interactions. Video data from each session will also be used to indicate the amount of explicit instruction (talking time of the trainer) and amount of music use, to validate the fidelity of the interventions. These data may be included as covariates in data analyses if they are significantly different across groups.

#### Secondary outcomes

EEG will be recorded using Epoc X (EMOTIV) research-grade EEG headsets with 14 channels digitized at 256 Hz. This tool is a child-friendly wireless mobile EEG system that is easy to apply with non-sticky saline-based wet sensors and has high research quality [16]. The data collection will be conducted by research assistants who are blinded to the participants’ group assignment. Recording will be performed in three conditions, namely, resting state, social scenes, and preferred music, for 5 min each. For the baseline resting state condition, data will be recorded with participants at rest, passively viewing an animation of moving shapes without sound. Although resting-state qEEG in adults is usually measured without explicit sensory stimulation, a simple viewing task is used here because it will be difficult for children to sit still without anything to hold their attention [16]. For the social scene condition, participants will view typical scenes where children or adults interact in school and playground without sound. For the preferred music condition, the participants will listen to their preferred music with eyes opened and fixated on a cross on a computer screen.

To determine the music preference of participants, a brief selection task of 25 different music pieces and genres will be administered, based on a task adapted from previous research reporting a five-factor music preference model called MUSIC [20]. Each music excerpt will be 15 s long, and participants will indicate their preference for each piece based on a 5-point pictorial scale. The dominant music style will be calculated for each participant and 5 min of preferred music pieces will be played to obtain neural responses to music in the pre-test and post-tests.

EEG segments free of artifacts will be selected, and spectrum decomposition will be carried out. Absolute and relative power levels in different frequency bands (alpha, beta, delta, gamma, and theta) will be calculated in each condition. FAA index will be calculated by subtracting the alpha frequency power from left and right frontal electrodes. The difference in FAA in the social scenes and baseline will be used as a predictor in data modeling in treatment and control groups. The FAA in preferred music condition will be correlated with treatment effectiveness in the treatment group only.

### Statistical analyses

Interim analyses will not be performed and data collection will continue until the intended sample size is reached. The investigators and research assistants will have full access to the final trial dataset. Analyses will focus on hypothesis-driven testing with separate models for each outcome measure by using linear mixed effect modeling if the assumptions for linear effects are met [21]. This regression-based statistical test can be used to evaluate multiple categorical and continuous predictors, and eliminates the need to run multiple tests for different hypotheses, which inflates the risk for type I error. Attrition bias will be assessed by comparing the participant completion rate in the two conditions and considering the reasons. Mixed effect models are robust with missing or extreme values, which is useful if the attrition rate for the 12-week intervention is higher than expected. Fixed effect predictors will be intervention type (music/non-music), time (pre−/post-intervention), and pre-treatment EEG frontal asymmetry (social and music). The random effects of participants and groups will be included to estimate random errors arising from individual differences in participants and group trainers. Control factors will include overall and verbal IQ, age, and ASD severity. The inclusion of these covariates alleviates concerns on unreliable effects due to the heterogeneity of children with ASD and ID.

## Discussion

In Hong Kong, a government policy was in place where special schools were provided with additional funding from the Education Bureau to run the Resource Teaching Program for children with low-functioning ASD. This program was particularly designed for those students with comorbid ID and ASD to receive enhanced support in addition to normal classroom teaching. The program was delivered in the form of individual or small group intervention, in-class support and follow up intervention [22]. If the results of the proposed study are positive and mechanisms in which music can promote social behaviors can be identified, then music can be a low-cost and scalable method to integrate social skill intervention in specific programs, regular school curricula, or as after-school activities.

Critically, if pre-treatment neural markers can help identify appropriate intervention plans, then application of this result will greatly improve the efficiency of treatment and educational provision for this population. EEG data can be collected with low-cost, commercially available products and take only 5–10 min to set up. Children with ASD and ID are only required to sit still for a few minutes because no explicit task response is needed. With the relative ease of data collection, this procedure may be included in routine clinical check-ups. The analyses of these data can help researchers and clinicians to understand how neural responses in children with ASD and ID are related to the clinical presentation of their social deficits. Such data may be further compared with those of neurotypical children to elucidate the mechanisms of social functioning and to improve social skill interventions. Individualized help in social skills for at-risk school-aged children may reduce cases of bullying and increase the likelihood of inclusion into the society. Downstream benefits may include increased success in securing and maintaining jobs and increased mental health for individuals and their caregivers.

The current lack of treatment standards has often led to confusion and stress for families with children with ASD. Public intervention services for children with ASD are not widely available and private services are expensive. In 2020, the Jumpstart program at Autism Partnership, the primary organization providing intensive ABA intervention for individuals with ASD in Hong Kong, cost HK$86,900 for 5 days of individual ABA therapy [23]. The Accelerate Program, which was delivered in small groups, was priced at HK$128,000 per month [23]. The Sparks program, which was a low-cost alternative of one-on-one therapy with relatively inexperienced therapists, still cost HK$32,500 per month [24]. For reference, the median household monthly income in Hong Kong in the second quarter of 2020 was HK$25,500 [25]. Given the cost considerations, some families may abandon treatment if the method is not effective in a short period of time. Prediction of treatment success may solve an urgent issue for families with children with ASD because the window for early intensive intervention is small, and the costs for different types of interventions are high. A weekly group-based music therapy session as described in this study costs approximately $500 per month. If music therapy is a reliable option to improve social outcomes in certain individuals with ASD and ID, then it may be a more cost effective alternative than intensive behavioral therapy.

The proposed study’s results may suggest directions to manpower planning and provision of services in Hong Kong, which may include policymaking and development of education and training programs at local tertiary institutions. Musical intervention in Hong Kong is increasing in popularity relative to other places that already routinely use music therapy in allied health professions. Practicing music therapists in Hong Kong mostly received training from Australia, Canada, the UK, or the USA. Although specialized clinical training and professional regulations are needed for music therapy practice, no local institution provides training that will lead to a recognized music therapist status, and no licensing organization exists in Hong Kong. If empirical data support the use of music therapy to increase social skills in children with ASD and ID, then the development and training of professionals to engage in such evidence-based practice may be particularly beneficial for Hong Kong.

Findings will be disseminated through presentations at scientific conferences, meetings for local practicing professionals in special education, and knowledge exchange seminars for the public and families of individuals with ASD and ID. Results will be reported in manuscripts to be submitted to relevant academic journals. The full protocol, anonymized participant-level dataset, and statistical codes will be made publicly available after study completion and report.

## Data Availability

Not applicable.

## Abbreviations

ASD: Autism spectrum disorder
CARS-2: Childhood autism rating scale-second edition
FAA: Frontal alpha asymmetry
ID: Intellectual disability
NGO: Non-governmental organization
qEEG: Quantitative electroencephalogram
RCT: Randomized controlled trial
SRS-2: Social responsiveness scale-second edition
